# Towards defining quality in home care for persons living with dementia

**DOI:** 10.1371/journal.pone.0274269

**Published:** 2022-09-13

**Authors:** Marianne Saragosa, Lianne Jeffs, Karen Okrainec, Kerry Kuluski

**Affiliations:** 1 Institute of Health Policy, Management and Evaluation, Dalla Lana School of Public Health, University of Toronto, Toronto, Canada; 2 Sinai Health, Toronto, Canada; 3 Lunenfeld-Tanenbaum Research Institute, Toronto, Canada; 4 Division of General Internal Medicine, University Health Network, Toronto, Canada; 5 Institute for Better Health, Trillium Health Partners, Mississauga, Canada; University of Ottawa, CANADA

## Abstract

**Introduction:**

Accelerating rates of dementia worldwide coupled with older adults living longer in the community calls for greater focus on quality home care support services. Few frameworks for quality dementia home care exist though prior findings have found elements considered to be important for “good” home care for people living with dementia. This study aimed to identify core components of a quality home care experience for people with dementia and their caregivers.

**Methods:**

As part of a larger research study, in-depth interviews were conducted with persons living with dementia and caregivers (n = 25) to explore hospital-to-home care transitions. The design used for this study was a qualitative description. We used deductive-inductive thematic analysis, which was informed by previous work in this area. Open codes were mapped to pre-determined themes, and for codes not accommodated by an *a pior*i framework, new themes were developed.

**Findings:**

Our findings resulted in 4 overarching themes. Two themes were identified deductively (Availability and Acceptability of Home Care Services) and two inductively (Adaptability and Affordability of Home Care Services). Findings highlight the roles of family—care provider partnerships and responsive support in receiving quality home care, and the cost associated with unmet needs.

**Interpretation:**

With an aging population, an increase in home care client acuity, and post-COVID-19 concerns over long-term care, more attention is needed to improve the quality of home care. The demand for these services will continue to increase particularly for those living with dementia and their families. The findings of availability, acceptability, adaptability, and affordability as core to quality care can help lay the groundwork for a home care framework for persons living with dementia and their caregivers. Future research could benefit from comparative analyses to evaluate the applicability of the findings to non-dementia home care service users and caregivers.

## Introduction

Accelerating rates of dementia worldwide is a significant challenge to health systems [1]. In Canada, 500,000 people are living with dementia [2]. By 2031, this figure is projected to almost double to 937,000 [2]. As the majority of older adults have a desire to age at home, including those living with dementia, higher reliance on home and community care support services is expected to increase [3]. Home care supports and services are designed to enable older adults to safely remain in their own homes by providing a suite of support services, like nursing, personal support, and rehabilitation [4] Compared to community dwellers without dementia, those with dementia are more likely to use home care support services over time [5,6]. Further, individuals affected by dementia and their caregivers were particularly vulnerable to restricted or suspended access to services including home care amid the COVID-19 pandemic [7,8]. Notably, though home care service utilization declined dramatically during the first COVID-19 wave (March and April 2020), home care service utilization surpassed historical levels by end of September 2020 [8], signalling an even greater demand in the home care sector.

Due to the progressively debilitating and dependency provoking nature of dementia, family caregivers provide the bulk of support. Canadian family caregivers give 26 hours of support on average per week compared with 17 hours to older adults who do not have dementia [9]. Access to formal home care can support caregivers in their role in helping to manage the daily challenges of providing care for persons living with dementia, care, can even delay entry into long-term care (LTC) [10,11]. Home care is designed to help support people in their own homes, rather than in hospitals or LTC, and for them to live as independently as possible [4]. For people with ongoing care needs, home care may be required following a transition from the hospital. A recent study centered on care transitions identified the quality of home care as central for patients and family caregivers [12]. Challenges included accessing a range of needed community services and dealing with untimely, insufficient, and unreliable home care services [12]. Dissatisfaction with service quality, notably the lack of responsiveness and flexibility to the holistic needs of patient-caregiver dyads, was found in the international caregiver literature [13]. Poor quality of in-home care is also a risk factor for LTC placement [14].

In contrast, studies have identified elements considered to be important for “good” home care for people living with dementia [11,15–17]. These elements include the availability of formal home care services and support, in addition to the perceived knowledge, security and dignity needed to care for a person with dementia [11,15]. While few frameworks for quality dementia home care exist for use in general health settings [18], Forbes and colleagues (2008) previously identified the *availability* and *acceptability* of formal services as central to positive home and community care experiences. However, absent from the literature is a recent overarching understanding of what defines quality home care and associated best practices for those living with dementia in the community. A better understanding could help to improve models of care, workforce training and quality standard development of home-based care to support people with dementia and their caregivers. Therefore, the objective of this study was to identify core components of a quality home care experience as perceived by persons living with dementia and their family caregivers.

## Design and methods

### Design

A qualitative study was undertaken to capture the hospital-to-home care transition experience of older adults living with dementia and their family caregivers. A series of in-depth semi-structured interviews were conducted by an experienced qualitative researcher who also has clinical experience as a nurse in the areas of mental health and geriatrics. The participants were not previously known to the researcher and the researcher established rapport during the consent and interview process. To capture the perceived quality of home and community care supports, the design used for this study was qualitative description. COVID-19 restrictions placed on in-person recruitment and data collection limited access to potential participants for face-to-face interviews. Instead, we developed and drew on pre-existing relationships with stakeholders (i.e., clinicians, caregiver advocates) and organizations (senior support services, dementia support groups) in Canada to support the dissemination of study material for recruitment purposes. Since the bulk of data collection occurred during COVID-19, most interviews happened over a videoconferencing platform (i.e., Zoom) apart from three in-person interviews before the pandemic. Virtual data collection facilitated researcher—participant rapport building by way of seeing each other and responding to nonverbal cues [19]. Many recruited caregivers identified as previously actively caring for someone living with dementia who had since passed away or transitioned to LTC due to an advanced stage of the disease.

### Sample

A purposeful sampling technique was used to identify participants initially from the hospital setting and then the community following COVID-19 restrictions. Inclusion criteria for participating were people aged 65 or older: who had a diagnosis of dementia at a mild to moderate stage, who had at any time experienced a transition from hospital back to their home; and/or a family caregiver, who cares or previously cared for a person living with dementia who had experienced a transition in care at any time from a hospital back to their home. Participants were not limited to a geographic area in Canada and a diagnosis of dementia was determined based upon self-report of a diagnosis. The interview guide consisted of open-ended questions about the care transition experience, including a focus on supports and resources available following the discharge home (see Box 1 for sample interview questions). Demographic data were collected before the interview. Interviews occurred between December 2019 and October 2021, lasted on average 67 minutes, and ranged between 38 and 99 minutes. All interviews were digitally recorded and transcribed verbatim. Interviewing ended on reaching thematic saturation, which we identified as the point when no newly identified codes or themes emerged from the final interviews.

**Box 1. Sample interview questions**

**Exploring the Transition from Hospital to Home**

1. Tell me about a hospital admission and transition back home that you experienced…
  - *Probe*: As you look back on your stay in the hospital, how were you prepared for the transition home?
  - *Probe*: How were you involved in the discharge planning?
2. Tell me about your most recent transition home from [hospital name]?
  - What was it like for you [caregiver]?
  - What role [caregiver] did you play?
  - Who, if anyone, influenced your experience? [probe: ask them to elaborate, how was their experience influenced, etc.]
3. What needs did you have during the transition process?
  Probe: Educational, informational, relational, management
  - How were these needs met or not met?

**Exploring the Adjustment to Being at Home**

4. Can you tell me more about your role as [NAME OF PATIENT]’s caregiver?
5. What was life like once you returned home from the hospital?
6. Did your role as caregiver change for [NAME OF PATIENT] once he/she returned home?
  Probe: How were you prepared? How did you feel?
7. Tell me about coordinating care since being discharged?
  Probe: How has it been arranging appointments, setting up and accessing services?

**Discussing Supports and Recommendations**

8. What were some resources or supports that you were able to access?
9. What, if anything, would have made things easier (e.g., family/friends, community, medical)?

### Analysis

The analysis closely followed Braun and Clarke’s thematic process [20], and it incorporated an inductive-deductive approach. Once the transcribed data was read and re-read (Phase 1), for Phase 2, initial codes were generated inductively by the first author (MS) across the entire data set. Phase 3 involved re-focusing the initial codes by mapping relevant codes to themes derived from Forbes et al.’s definitions of ‘Availability’ and ‘Acceptability’ of formal care services for persons with dementia and their family caregivers (see Table 1 for Forbes et al.’s themes/sub-themes and definitions). Following the collating of inductive codes to the deductively identified themes, themes were further refined (Phase 4). For example, reviewing and reworking the themes created several new themes [20]. While the analysis utilized a priori concepts and their definitions, new themes were also created inductively where data were not accommodated by the framework to avoid forcing data into prescribed categories [21]. Rigor was determined using a set of strategies including a coding system, and peer debriefing with other researchers and clinicians [22,23]. Engaging in memo writing and reflexive practice also served as a way of establishing rigor [24].

**Table 1:** Forbes et al, themes, sub-themes and definitions.

Themes and Sub-themes	Definition
***Availability of Home and Community-Based Services***	Refers to the extent to which participants had access to services that could help caregivers in their care work and enable the person with dementia to remain in their own homes.
***Acceptability of Home and Community-Based Services***	Perceived quality of service and relevant dimensions are noted as the sub-themes below.
***Comprehensive Assessments*,** ***Treatments*, *and Provision of*** ***Dementia Care***	Receipt of comprehensive, thorough assessment through engaging with professional support.
***Inconsistency of Care*** ***Providers***	Breakdown in the continuity of care by the provision of inconsistent care providers and delivery of services.
***Attributes of Trusting*** ***Partnerships***	Challenges establishing trusting partnerships based on inconsistent care providers, perceived providers being insensitive to the needs of family members, and not meeting expectations of professional conduct.
***Inflexible Care***	Lack of customizable planning to meet individual client needs.
***Cost of Services***	The fee associated with supportive services (e.g., homemaking assistance)

### Ethical approval

Ethical approval and subsequent amendments to the study design were granted by Sinai Health’s Research Ethics Board and the University of Toronto’s affiliated Research Ethics Board. For persons living with dementia who could not provide written informed consent, consent was obtained from a substitute decision maker. In interviews that happened in-person, ongoing assent (or dissent) was observed during the interview process [25]. When signed consent was not possible due to COVID-19 restrictions, we received and recorded verbal informed consent before the interview starting from every study participant.

### Findings

Twenty-one interviews happened with twenty-five participants: 4 persons with dementia with their caregiver (spousal = 3, daughter = 1), and 17 caregivers individually. Most participants were female (n = 17, 68%), were spouses of the people they were caring for (n = 10, 47%) or children (n = 9, 43%). The most common employment status of the caregivers was “retired” (n = 12, 57%), followed by “employed for wages,” (n = 5, 24%). Most caregivers relied on a mix of public (n = 20, 95%) and family assistance (n = 14, 66%), and a lesser amount had private help (n = 6, 28%) (see Table 2).

**Table 2:** Participant demographics.

**Participant Characteristics**	
**Persons with dementia (n = 4)**	
**Gender (n)** Female Male	1 3
**Age, Years (mean, range)**	80.75, 68–91
**Duration of dementia diagnosis, Years (mean, range)**	9.75, 3–16
**Education completed (n)** Less than high school Graduate education	2 2
**Family caregivers (n = 21)**	
**Gender (n)** Female Male	16 5
**Age, Years (mean, range)**	63.67, 21–94
**Duration of caregiver role, Years (mean, range)**	7.66, 1–22
**Relationship status, n** Spouse Child Daughter-in-law Granddaughter	10 9 1 1
**Employment status, n** Retired Employed for wages Student Out of work and looking Unable to work	12 5 2 1 1
**Additional sources of support** Public Private Family Friends	20 6 14 2

Our analysis resulted in two themes framed by Forbes, Markle-Reid (26) findings that speak to components of a quality home care experience for people with dementia and their caregivers. We also present two additional themes.

### Availability of home care services

Availability of home care services refers to caregivers perceiving access to services that help in their care work and support the person with dementia [26]. These services ranged in type by assistance with daily living (i.e., bathing, medication administration), in-home respite, home modifications, support with physical exercise, and palliative care. They also varied in meeting the needs of families from being fully accessible, to services being described as unavailable yet needed. To address gaps in service, families pursued private help or LTC placement.

In response to the older persons with dementia experiencing a change in health status that required hospitalization, family caregivers described being referred to home care services. Several mentioned seeing either an occupational therapist (OT) for a home safety assessment or a physical therapist (PT) for physical exercise. Family caregivers often reported minimal benefit from the involvement of publicly funded OTs and PTs mostly because these providers couldn’t suggest additional home improvement ideas, their involvement was considered not long enough, and caregivers sensed a lack of coordination between the service providers:

*“It was so frustrating even dealing with the OT that we were assigned through the [agency]*. *She never came to the house*. *It was all done over the phone*, *and she had no idea of what to suggest for bed alarms*, *bed mats*, *anything like that*.*”*

*P019 Daughter caregiver*

Access to home care services was paramount in allowing caregivers respite, or an “occasional break.” Respite occurred through publicly funded in-home services or a day program. During this timeframe, the needs of the person with dementia were momentarily attended to by service providers. Caregivers described the respite as essential to their capacity to care, as a form of self-care, and a time when they “wouldn’t have to think about so many things” (P016, spousal caregiver). For a few caregivers, an absence of respite services resulted in feeling physically and mentally unwell, and even social withdrawal,

*“I said I haven’t had a holiday since 2010*. *Except for home care providing 2 days of respite once*. *So it’s very hard on you*. *Socially*, *you lose all your friendships*. *All your ties*.*”*

*P020 Daughter caregiver*

In addition to respite, caregivers described challenges of not having enough home care hours, and not being able to access or not knowing what services were available to them. With inadequate hours of care, caregivers were not able to pursue paid work or complete essential tasks, like picking up groceries because the person they were caring for required constant supervision. Gaps in care for those with complex physical and behavioural needs meant poorer outcomes. Notably, skin breakdown, depressed mood and premature transfer to LTC care were frequently mentioned. In the former cases, private personal support workers (PSWs) hired by the family were used to “fill in” and help manage where the public services fell short,

*“We just weren’t prepared at all*, *and if it hadn’t been for this PSW agency*, *I don’t know what we would have done*. *Because even when I got home as they would–I was paying–they’d like to make her meals*, *like take her for a walk*, *like whatever*, *while we just took care of my dad*.*”*

*P008 Daughter caregiver*

The COVID-19 global pandemic amplified the challenges caregivers and people with dementia had with the availability of home care services. Restrictions put in place had one caregiver concerned about service withdrawal and limits around hiring private help. Feelings of abandonment surfaced when she acknowledged that she couldn’t access respite:

*“I can’t go out and have a coffee because I might bring it [COVID] home with me*. *And then*, *you can’t hire any respite*. *The homes have clamped down*, *the personal care workers*, *homecare is clamped down*, *they’ve really just abandoned–like I do believe that they have abandoned a segment of the population*. *So I think I’m just lucky enough to have made it this far*.*”*

*P004 Daughter caregiver*

### Acceptability of home care services

Acceptability of home care support services is defined as the perceived quality of care by service users [26]. This theme has three sub-themes: receiving quality dementia care, consistency in provider and scheduling, and trusting partnerships.

#### Receiving quality dementia care

*Quality* of dementia care is defined by caregivers in this study as home care service providers who were knowledgeable, well-trained, and shared relevant and useful information. Frequently, caregivers interacted with home care support providers and detailed both positive and challenging situations. When dementia care was described positively, the needs of the individuals were seen as being met by trained and compassionate individuals. For example, two PSWs provided “excellent care” to a person with dementia because they personalized the care experience:

*“They gave me two men to come and they were excellent*. *I would take the morning off because they would feed him and by then they both knew him*, *and I had picked good people*. *They could take my husband out down the stairs and put him up in the sling*.*”*

*P005 Spousal caregiver*

Similarly, family caregivers noted when service providers related well to the person with dementia: “One of them was really good. She could have at least distract her with something else, get her to tell stories about herself, that’s always good for half an hour or 45-minute distraction, she could do that, (P017, daughter-in-law caregiver).

Caregivers also reported concerns with the level of training among providers in the home care setting. In the home environment, family members recognized deficits in knowledge or training, and offered support to the service provider: “It was very hard and some of them [PSWs] were not really trained to deal with dementia patients. So, we had to train them and we had no training ourselves” (P018, daughter caregiver). Caregiver participants often advocated for service changes, or compensated by taking on certain tasks themselves:

*“So*, *I decided I would try and move him on my own*, *so I bought some slide sheets and things*. *And of course*, *I couldn’t do it on my own…it would have been very dangerous*. *Both of us could’ve been hurt because he’s dead weight*.*”*

*P015 Spousal caregiver*

#### Experiencing scheduling and care provider consistency

A dominant experience across many of the caregivers that were interviewed was both inconsistent providers and scheduling of home care services. The following scenario stood out as the exception when one spousal caregiver acknowledged consistency in PSWs, scheduling and a quick resumption of services following the hospital discharge.

*“We already had our services set up with the [community care coordinator] and we’ve been told that*, *although they were discontinued*, *they would restart as soon as she came home*, *and see*, *she came home Tuesday and XName [PSW] was here Wednesday of last week*.*”*

*P001 Spousal caregiver*

Family caregivers expressed frustration over not having the same person or having care delayed by several hours. During the COVID-19 pandemic according to several participants, the same providers were rarely scheduled. In response, they felt with staff changing often, they had to “start from the beginning” (P011) or that they “had no say” (P013) in how care was being delivered. Caregivers were unsure what to do when care was cancelled,

*“They [PSW agency] would call at 08*:*00 in the morning with someone saying*, *‘Sorry we’re not coming and it’s like*, *‘Yeah thanks for letting me know but what am I supposed to do*?*’”*

*P015 Spousal caregiver*

For one caregiver, having no reliable home care schedule or consistent provider seemed to be “more disruptive” (P019). Lastly, another point of frustration was when home care services were unnecessarily split up either by agencies or by the timing according to caregivers, which amplified inconsistent providers throughout the day:

*“They were coming in one hour for [Name] and coming back two hours later to give me my respite*. *Well I mean poor [Name]*, *he knows himself that the service is supposed to be combined*, *but they don’t have the policy in place to say well this is how it’s supposed to be done*. *You’re not supposed to be doing it that way because it doesn’t make good dementia care*.*”*

*P013*, *Spousal caregiver*

To address the challenges with the consistency of publicly funded care, caregivers opted to hire private PSWs to “balance off” the perceived unpredictability of home care. These inconsistencies and scheduling gaps also contributed to increased care provision by caregivers,

*“There was a period there where I was on duty a lot more than normal*. *There were times when PSWs*, *in conjunction with the staff or their agencies*, *weren’t coming in because they were told not to come in*, *so I had to deal with everything*.*”*

*P003 Spousal caregiver*

#### Recognizing collaborative partnerships

One factor that helped to mitigate poor home care services was collaborative working relationships among the person with dementia—family caregiver—provider triad. Exclusionary practices, like not involving caregivers in decision-making or service planning, were noted to negatively affect family—provider alliances. Family caregivers described occasions of advocacy for improved communication or observed greater support at the provider level.

Collaborative relationships emerged in the form of caregivers and health providers across the care continuum (i.e., hospital and community) working in partnership to support effective transitions. In several instances, hospital and community-based care coordinators worked together to assist families,

*“The [community] worker stepped in*, *probably beyond what she was allowed to do but she helped the worker at the hospital sort it out so that we could get appropriate care immediately upon leaving the hospital*.*”*

*P014 Daughter caregiver*

Family caregivers not only described these synergistic connections, but they mentioned their long-standing relationships with community-based care coordinators who listened to and understood their needs. When these relationships were not present, and system navigation and care plan discussions were not addressed, caregivers expressed feeling unsure about what was happening and felt excluded.

Several caregivers acknowledged when community-based care coordinators “went to bat” for them, or advocated for the needs of the person with dementia and their caregiver. One spousal caregiver noted that the success of a discharge from the hospital was dependent on who supported the family. While some participants had a similar sentiment about advocacy at the community provider level, a few noted that they were left feeling like they had no say. The following quote illustrates competing tensions between a family caregiver and service provider,

*“Because of all of the issues that the crises brought up in the house*, *the [community service provider]’s suggestion was to escalate him to long-term care and at the end that’s where he went…So it was a very frustrating experience because no matter how much I tried to advocate*, *tried to put things in place in the house*, *I felt that we were sunk from day one*. *And this was a direction they [community care coordinator] felt this was going to go and there was nothing we could really do about it*.*”*

*P019 Daughter caregiver*

### Adaptability of home care services

In our study, adaptability of home care services was identified as the perception of service adjustment made in response to not only the shifting needs of the person with dementia but also caregiver needs as well.

Service adaptability is noted in several examples and involved geographical service transfers, more hours of care, and additional personal support in the home. In two cases, a sharp increase in patient acuity called for more help, which caregivers requested and received. The following is one such example that represents the adaptability of service provision to meet client and caregiver needs:

*‘‘He’s coming home so the [care coordinator] person said to me*, *‘What do you need*? *Like what do you think the hours you would need’ so I picked a number in the air and I kind of said*, *you know*, *two hours*, *two hours–so we ended up at 10 hours a day*. *OK*, *so they went to work and so the next thing they say*, *‘OK*, *we’ll give you 70 hours a week’*, *like OK*, *I’ve never heard of this but*, *hey*, *I’ll take what I get*.*”*

*P011 Spousal caregiver*

Family caregivers seemed to also expect adaptability when they reported having contact information if they encountered an issue with service providers,

*“I have her number whenever there’s a problem*, *if a PSW doesn’t show up*, *even on the weekends I can get someone*, *so they’re very punctual and available if you have any problem at all*.*”*

*P001 Spousal caregiver*

Conversely, inflexible service response occurred when home care service was withdrawn unexpectedly, reduced with no plan in place, or was not able to accommodate specific scheduling requests. The service reduction transpired during the COVID-19 pandemic. It also meant added care responsibility for a caregiver that was already working full-time,

*“But then particularly during Covid*, *they would actually rather have you administering all three doses then*, *right*? *And that gets really complicated because that’s almost like–and I’m not trying to sound rude*, *but that’s almost a full-time job*, *right*? *And I have a full-time job*.*”*

*P0004 Daughter caregiver*

### Affordability of home care services

Perceived deficits in availability and acceptability in home care services drove many family caregivers to hire private help. However, this option was not possible for everyone because of their financial situation. For one family, relying on private personal support came at a steep cost,

*“I think that summer alone just*, *from the time my mom was with us I spent like $25*,*000 [CAD] in PSW charges*. *We were paying $10*,*000*, *$12*,*000 [CAD] a month so someone could sit with my mother for ten hours while we could spend time with my dad*.*”*

*P008 Daughter caregiver*

In regions that have a co-payment model associated with home care support, even $50 per visit was felt to be too much,

*“But when you [multiply] 50 bucks by two or three times a week*, *even just two times*, *that’s $100 a week*, *it’s $400 a month*, *it’s $4*,*800 a year*. *When you take that out of a small pension it gets very difficult*.*”*

*P012 Spousal caregiver*

Access to additional help enabled caregivers to complete essential tasks, like grocery shopping, attending their medical appointments, and continue working.

Many caregivers also described the impact of being a caregiver on their paid employment. Notably, early or forced retirement, a reduction in work hours, or a temporary leave of absence from a job were all reported. Parallel caregiving and working were sources of stress for caregivers since inadequate availability of service resulted in a reduction both in income and quality of life. Although one caregiver with her experience in dementia care saw an opportunity to pivot to a different career in housing design and adaptation. In this quote, an adult child caregiver expressed layers of loss when he retired early to care for his parents:

*“In 2017*, *when I retired to take care of my parents*, *I lost that sense of purpose*. *I didn’t understand how hard that would hit me at the same time*. *Taking care of my parents and losing my sense of purpose*, *like losing my sense of self*.*”*

*P007 Son caregiver*

## Discussion

Our study findings make several important empirical contributions. From an academic perspective, we confirm previous evidence that supporting people with dementia in the community requires *available* and *acceptable* home care services [16,26]. Findings also build on extant literature using recent accounts overlayed by the global pandemic to reveal significant gaps in the *adaptability* and *affordability* of home care services for our study population. Taken together, the study depicts an overarching understanding of what defines quality home care services categorized according to these four themes. Several key theoretical contributions are made that further highlight differences from Forbes et al.’s (2008) [22] earlier work and that is the role of family—care provider partnerships in receiving quality home care, an increased focus on adaptable systems, and the cost associated with unmet need. Taken together, the results suggest that a consistent and responsive home care system that does not contribute to the economic burden of unpaid caregiving is paramount to quality community-based care for people living with dementia. All four themes are discussed below in more depth.

The types and availability of home care services centred on personal support with daily living activities (bathing, medication administration, eating), respite care and to a lesser degree, OT, and PT involvement. Reliance on these support services was high in this sample, which is representative of the population living with dementia [27]. Other studies have shown extensive home care use among those living with dementia at a rate between 22% and 27.5% of all home care clients in Ontario [28,29]. The demand for home care is suggestive of both the affected individual and their families desire to stay in their own homes and the care needs of these individuals as being chronic and complex. For example, one study showed that 50.6% of people diagnosed with moderate to severe dementia living in the community were identified as “fully dependent” with moderate to severe “frailty” and support needs in many areas; food preparation, medication administration, falls prevention and looking after the home [30]. However, our findings with regards to gaps in home care availability are in line with not only the prior work of Forbes et al. (2008) but more recent evidence of unmet home care needs of this population [31–33]. Unmet home care needs were exacerbated by the response to COVID-19 when home care services were restricted or reduced, particularly during the early phase of the pandemic. Personal care and therapies for persons with dementia were significantly interrupted, although, home care services rebounded to historical highs [8,34]. Consistent with the literature [35–37], limits on home care service and family availability were associated with LTC placement. In our findings, regular use of respite was vital to caregivers’ capacity to care for and support their family members at home. Receiving regular breaks through respite care and community day programs have been identified as practices that help keep a person living with dementia in the community longer [3]. Although barriers to the use of respite services exist, which was seen in some caregivers not being able to access these services. Barriers to the use of respite by caregivers of people with dementia include not knowing where to find information, poor quality dementia care, and caregivers’ feelings of guilt, which lead to underutilization of in-home and adult day program respite services [38,39].

When people with dementia and their caregivers have access to home care services, their acceptability is often dependent upon the perceived quality of the care, provider and scheduling consistency and collaborative partnerships. Caregivers expressed the poor quality of care with formal care providers due to their perceived inability in managing dementia-related problems. Informal caregiver burden is well documented in the dementia literature [40], as is the increase of burden with poor quality and lack of continuity of care [41]. Conversely, knowing and relating well to persons with dementia were indicators of quality care. Recent study findings found that PSWs can identify person-centred approaches to care as essential to quality dementia home care; however, time constraints, task-oriented models of care, and limited training make it challenging to deliver a high standard of care [16,42]. Simply increasing home care hours without investing in a system and cultural change—embracing tenets of personhood, increasing PWS wages, and providing dementia-specific education and quality standards—will not result in better outcomes [16,43,44]. Similarly, previous findings echo frustration over inconsistent scheduling and staffing [17]. Furthermore, delivering person-centred care becomes even more challenging since relational discontinuity prevents meaningful personalization of care [41]. Unlike other studies that saw few case managers take on an active advocacy role [32], our study findings revealed novel insights around strong family—care coordinator collaborative partnerships that fostered trust, and when not present, caregivers were left with a sense of disempowerment. The present study illuminated that the lack of quality home and care services could be mitigated by relational continuity from their care coordinator, or another trusted health care provider. *Relational continuity* is defined as having at least one, most trusted provider among many who has a comprehensive knowledge base of the patient and uses that to inform the management plan [45]. Some attention has been paid to relational continuity in dementia and home care literature. Relational continuity contributes to a lower risk of Emergency Department presentation and hospital admission [46], improvement in the quality of home health care [47], and it is essential to collaboration between formal and informal care providers [48].

The following theme of *adaptability* builds on Forbes’ sub-category of “inflexible care” (p. 90).

Acute changes in the person’s condition, like rapid functional decline or events like COVID-19, warranted adaptable and flexible home care service delivery. Yet, the extent to which services were flexible varied and was determined more by the care coordinator than the family caregiver. Study findings align with what was found in Forbes et al.’s earlier work on inflexible care as contributing to dissatisfaction with care and perceived burden among family caregivers [26]. PSWs have also identified adaptability and flexibility as core components of high-quality home care for persons with dementia [16]. The tension between what is considered essential and valued in quality home care, like personalized and flexible care, is at odds with constraints imposed by the existing home care system [49,50]. For example, home care case managers tend to pay more attention to the lack of available resources or system pressures when making care decisions while family caregivers consider the relational effects that decisions would have on the family unit [50]. Canadian literature has identified variable resource allocation in home care and the factors at play, including differences in the older client and caregivers themselves, as well as the discretion and flexibility based on professional training and insight [51]. This is consistent with “street level bureaucracy” theory that says extreme discretion by frontline professionals is used to make or adapt the policy to client needs and circumstances [52]. Such discretion can be thought of as inequitable, inadequate, or inconsistent, which may lead to sub-optimal or discrepant decisions across cases [51].

Study findings revealed the direct cost associated with inadequate levels of home care support services for people with dementia and their families. Early retirement, loss of wages and lost productivity due to the burden of care were also seen as the indirect costs of caring. In the previous availability and acceptability of home care paper, Forbes et al. (2008) briefly reported on the “cost of services.” Our results draw insight from this sub-theme to identify affordability as the final theme. Since the majority of those living with dementia are cared for in their own home by family, the economic burden falls on families rather than the health system. Data suggests informal costs (amount of informal caregiver’s time provided for care) are the main cost drivers at 60 to 84%, followed by medication costs and direct non-medical costs (paid help or transport) [53]. Family caregivers take on many unpaid tasks with little preparation, like supervision and training, care coordination, and building relationships [54], which effectively removes family caregivers from the workforce.

## Implications

Our study highlights the need for improved quality in the delivery of home care support services for persons with dementia and their caregivers. Such services would be expected to not only be available to those with dementia and those caring for them, but also acceptable, adaptable, and affordable. The findings indicate the following as central to quality care: that the care relationship is collaborative and adaptable to the changing needs of both the individual and their caregiver. Although, in contrast to other chronic conditions, dementia is perceived as a more challenging condition to manage [55]. People with dementia and their families also rely heavily on a support network of social and health service resources. Negative attitudes about dementia and inadequate support could make those with dementia more vulnerable to excessive disability and premature LTC placement. We argue that strengthening home care means adopting a radical shift in how persons with dementia are regarded. Sutherland and Wiersma (56) outline three values as key to any discussion that involves dementia policy, strategy, or change: the rights of people with dementia, diversity and equity, and inclusion, all framed by a citizenship lens. Therefore, to implement these components to quality home care, including person-centred care approaches, we need to first recognize people living with dementia and their families as citizens with rights [56].

## Limitations and strengths

The use of preconceived definitions of quality home care services by Forbes et al. (2008) enabled clear delineation between themes. Drawbacks to the analysis include a deductive analytical approach that might potentially force data into the a priori codes and having only one coder code the data. Although, input from different researchers provided greater rigour to the interpretation process. Since the data set coded was part of a broader qualitative study, the current study was not designed to be representative of a wider home care population. Considering this limitation, it may be relevant for future research in this area to focus on comparative analyses of ethnic monitories and non-dementia community-based care recipients and caregivers to assess the applicability of these themes. Importantly, the absence of firsthand accounts from persons living with dementia calls for future work that seeks knowledge of how people with dementia experience home care services in the Canadian context. This would assist in designing services that consider preferences, needs, and the tailoring of service to the individual with dementia. From an applied health research perspective, our study design had the flexibility to develop a deeper understanding of quality dementia home care, and even the potential to advance the dementia home care quality agenda.

## Conclusion

With an aging population, increase in home care client acuity, and post-COVID-19 concerns over LTC, more attention is needed to improve the quality of home care. The demand for these services will continue to increase particularly for those living with dementia and their families. The findings of this study can help to lay the groundwork for core components of quality home-based dementia care—availability, acceptability, adaptability, and affordability. The interplay between these four components is foundational to informing quality standards and related strategies to enhance care delivery with attention paid to the needs of people living with dementia and those who care for them.

## Supporting information

S1 File (DOCX)

## References

[1] World Health Organization. Dementia A Public Health Priority. In: WHO, editor. nd.

[2] Alzheimer Society of Canada. Prevalence and Monetary Costs of Dementia in Canada. Toronto, ON: The Alzheimer Society of Canada, 2016.

[3] Canadian Institute for Health Information. Dementia in home and community care nd [cited 2022 January 21]. Available from: https://www.cihi.ca/en/dementia-in-canada/dementia-care-across-the-health-system/dementia-in-home-and-community-care.

[4] Government of Canada. Home and community health care 2016 [cited 2022 January 21]. Available from: https://www.canada.ca/en/health-canada/services/home-continuing-care/home-community-care.html.

[5] Bronskill SE, Maclagan LC, Walker JD, Guan J, Wang X, Ng R, et al. Trajectories of health system use and survival for community-dwelling persons with dementia: a cohort study. BMJ open. 2020;10(7):e037485. doi: 10.1136/bmjopen-2020-037485 32709654 PMC7380876

[6] Mondor L, Maxwell CJ, Hogan DB, Bronskill SE, Campitelli MA, Seitz DP, et al. The Incremental Health Care Costs of Frailty Among Home Care Recipients With and Without Dementia in Ontario, Canada. Medical care. 2019;57(7):512–20. 31107398 10.1097/MLR.0000000000001139

[7] Bacsu J-DR, O’Connell ME, Webster C, Poole L, Wighton MB, Sivananthan S. A scoping review of COVID-19 experiences of people living with dementia. Canadian Journal of Public Health. 2021;112(3):400–11. doi: 10.17269/s41997-021-00500-z 33825134 PMC8023523

[8] Jones A, Maclagan LC, Schumacher C, Wang X, Jaakkimainen RL, Guan J, et al. Impact of the COVID-19 pandemic on home care services among community-dwelling adults with dementia. Journal of the American Medical Directors Association. 2021;22(11):2258–62. e1. doi: 10.1016/j.jamda.2021.08.031 34571041 PMC8422852

[9] Canadian Institute for Health Information. Unpaid caregiver challenges and supports nd [cited 2022 January 21]. Available from: https://www.cihi.ca/en/dementia-in-canada/unpaid-caregiver-challenges-and-supports#:~:text=CIHI’s%20analysis%20finds%20that%20caregivers,caregivers%20of%20those%20without%20dementia.

[10] Annerstedt L, ElmstÅhl S, Ingvad B, Samuelsson S-M. An analysis of the caregiver’s burden and the" breaking-point" when home care becomes inadequate. Scandinavian journal of public health. 2000;28(1):23–31. 10817311 10.1177/140349480002800106

[11] Lindeza P, Rodrigues M, Costa J, Guerreiro M, Rosa MM. Impact of dementia on informal care: a systematic review of family caregivers’ perceptions. BMJ Supportive & Palliative Care. 2020. doi: 10.1136/bmjspcare-2020-002242 33055092

[12] Kiran T, Wells D, Okrainec K, Kennedy C, Devotta K, Mabaya G, et al. Patient and caregiver priorities in the transition from hospital to home: results from province-wide group concept mapping. BMJ Quality & Safety. 2020;29(5):390–400. doi: 10.1136/bmjqs-2019-009993 31907325

[13] Morrisby C, Joosten A, Ciccarelli M. Do services meet the needs of people with dementia and carers living in the community? A scoping review of the international literature. International Psychogeriatrics. 2018;30(1):5–14. doi: 10.1017/S1041610217001491 28784193

[14] McClendon MJ, Smyth KA. Quality of in-home care, long-term care placement, and the survival of persons with dementia. Aging & mental health. 2015;19(12):1093–102. 25634669 10.1080/13607863.2014.1003284

[15] Goh AM, Polacsek M, Malta S, Doyle C, Hallam B, Gahan L, et al. What constitutes ‘good’home care for people with dementia? An investigation of the views of home care service recipients and providers. BMC geriatrics. 2022;22(1):1–13. 35016640 10.1186/s12877-021-02727-4 PMC8751242

[16] Breen R, Savundranayagam MY, Orange JB, Kothari A. Quality home care for persons living with dementia: Personal support workers’ perspectives in Ontario, Canada. Health & social care in the community. 2021. doi: 10.1111/hsc.13692 34951066

[17] Polacsek M, Goh A, Malta S, Hallam B, Gahan L, Cooper C, et al. ‘I know they are not trained in dementia’: Addressing the need for specialist dementia training for home care workers. Health & social care in the community. 2020;28(2):475–84. 31646701 10.1111/hsc.12880

[18] Health Quality Ontario. Dementia Care for People Living in the Community nd [cited 2022 April 6]. Available from: https://www.hqontario.ca/portals/0/documents/evidence/quality-standards/qs-dementia-clinician-guide-en.pdf.

[19] Archibald MM, Ambagtsheer RC, Casey MG, Lawless M. Using zoom videoconferencing for qualitative data collection: perceptions and experiences of researchers and participants. International Journal of Qualitative Methods. 2019;18:1609406919874596.

[20] Braun V, Clarke V. Thematic analysis. 2012.

[21] Carroll C, Booth A, Leaviss J, Rick J. “Best fit” framework synthesis: refining the method. BMC medical research methodology. 2013;13(1):1–16. doi: 10.1186/1471-2288-13-37 23497061 PMC3618126

[22] Morse JM. Critical analysis of strategies for determining rigor in qualitative inquiry. Qualitative health research. 2015;25(9):1212–22. doi: 10.1177/1049732315588501 26184336

[23] Colorafi KJ, Evans B. Qualitative descriptive methods in health science research. HERD: Health Environments Research & Design Journal. 2016;9(4):16–25. doi: 10.1177/1937586715614171 26791375 PMC7586301

[24] Rettke H, Pretto M, Spichiger E, Frei IA, Spirig R. Using reflexive thinking to establish rigor in qualitative research. Nursing Research. 2018;67(6):490–7. doi: 10.1097/NNR.0000000000000307 30067583

[25] Slaughter S, Cole D, Jennings E, Reimer MA. Consent and assent to participate in research from people with dementia. Nursing Ethics. 2007;14(1):27–40. doi: 10.1177/0969733007071355 17334168

[26] Forbes DA, Markle-Reid M, Hawranik P, Peacock S, Kingston D, Morgan D, et al. Availability and acceptability of Canadian home and community-based services: perspectives of family caregivers of persons with dementia. Home health care services quarterly. 2008;27(2):75–99. doi: 10.1080/01621420802022548 18928206

[27] Home Care Ontario. Dementia and Home Care Advice on Ontario’s Dementia Strategy 2017 [cited 2022 March 30]. Available from: https://www.homecareontario.ca/docs/default-source/position-papers/dementia-and-home-care—march-2017—home-care-ontario-6.pdf?sfvrsn=8.

[28] Mondor L, Maxwell CJ, Hogan DB, Bronskill SE, Gruneir A, Lane NE, et al. Multimorbidity and healthcare utilization among home care clients with dementia in Ontario, Canada: a retrospective analysis of a population-based cohort. PLoS medicine. 2017;14(3):e1002249. doi: 10.1371/journal.pmed.1002249 28267802 PMC5340355

[29] Vu M, Hogan D, Patten S, Jetté N, Bronskill S, Heckman G, et al. A comprehensive profile of the sociodemographic, psychosocial and health characteristics of Ontario home care clients with dementia. Chronic diseases and injuries in Canada. 2014;34(2–3). 24991776

[30] Abreu W, Tolson D, Jackson GA, Staines H, Costa N. The relationship between frailty, functional dependence, and healthcare needs among community‐dwelling people with moderate to severe dementia. Health & social care in the community. 2019;27(3):642–53. 30402986 10.1111/hsc.12678

[31] Gilmour H. Unmet home care needs in Canada. Health Rep. 2018;29(11):3–11. 30485384

[32] Ward-Griffin C, Hall J, DeForge R, St-Amant O, McWilliam C, Oudshoorn A, et al. Dementia home care resources: how are we managing? Journal of Aging Research. 2012;2012. doi: 10.1155/2012/590724 22132332 PMC3205668

[33] Yakerson A. Informal family caregiver experiences with publicly funded home care in Ontario. Home Health Care Services Quarterly. 2021:1–11. doi: 10.1080/01621424.2021.2006849 34842061

[34] van den Bulck AO, de Korte MH, Metzelthin SF, Elissen AM, Everink IH, Ruwaard D, et al. In the eye of the storm: A quantitative and qualitative account of the impact of the COVID-19 pandemic on dutch home healthcare. International Journal of Environmental Research and Public Health. 2022;19(4):2252. doi: 10.3390/ijerph19042252 35206437 PMC8872342

[35] Robison J, Shugrue N, Porter M, Fortinsky RH, Curry LA. Transition from home care to nursing home: Unmet needs in a home-and community-based program for older adults. Journal of Aging & Social Policy. 2012;24(3):251–70. 22720886 10.1080/08959420.2012.676315

[36] Afram B, Stephan A, Verbeek H, Bleijlevens MH, Suhonen R, Sutcliffe C, et al. Reasons for institutionalization of people with dementia: informal caregiver reports from 8 European countries. Journal of the American Medical Directors Association. 2014;15(2):108–16. doi: 10.1016/j.jamda.2013.09.012 24238605

[37] Morton-Chang F, Williams AP, Berta W, Laporte A. Towards a community-based dementia care strategy: how do we get there from Here? HealthcarePapers. 2016;16(2):8–32. doi: 10.12927/hcpap.2017.25006 28332962

[38] Parker LJ, Fabius CD. Racial differences in respite use among black and white caregivers for people living with dementia. Journal of Aging and Health. 2020;32(10):1667–75. doi: 10.1177/0898264320951379 32819177

[39] Leocadie M-C, Roy M-H, Rothan-Tondeur M. Barriers and enablers in the use of respite interventions by caregivers of people with dementia: an integrative review. Archives of Public Health. 2018;76(1):1–11. doi: 10.1186/s13690-018-0316-y 30479766 PMC6249779

[40] Papastavrou E, Kalokerinou A, Papacostas SS, Tsangari H, Sourtzi P. Caring for a relative with dementia: family caregiver burden. Journal of advanced nursing. 2007;58(5):446–57. doi: 10.1111/j.1365-2648.2007.04250.x 17442030

[41] Dalgarno EL, Gillan V, Roberts A, Tottie J, Britt D, Toole C, et al. Home care in dementia: The views of informal carers from a co-designed consultation. Dementia. 2021;20(7):2261–77. doi: 10.1177/1471301221990504 33530737 PMC8564226

[42] Kamalraj P, Savundranayagam MY, Orange J, Kloseck M. Communication in home care: Understanding the lived experiences of formal caregivers communicating with persons living with dementia. International Journal of Older People Nursing. 2021;16(6):e12401. doi: 10.1111/opn.12401 34337872

[43] Walsh S, O’Shea E, Pierse T, Kennelly B, Keogh F, Doherty E. Public preferences for home care services for people with dementia: A discrete choice experiment on personhood. Social Science & Medicine. 2020;245:112675. doi: 10.1016/j.socscimed.2019.112675 31760321

[44] Di Gregorio D, Ferguson S, Wiersma E. From beginning to end: perspectives of the dementia journey in northern Ontario. Canadian Journal on Aging/La Revue canadienne du vieillissement. 2015;34(1):100–12. doi: 10.1017/S0714980814000531 25631706

[45] Haggerty JL, Roberge D, Freeman GK, Beaulieu C. Experienced continuity of care when patients see multiple clinicians: a qualitative metasummary. The Annals of Family Medicine. 2013;11(3):262–71. doi: 10.1370/afm.1499 23690327 PMC3659144

[46] Jones A, Bronskill SE, Seow H, Junek M, Feeny D, Costa AP. Associations between continuity of primary and specialty physician care and use of hospital-based care among community-dwelling older adults with complex care needs. PloS one. 2020;15(6):e0234205. doi: 10.1371/journal.pone.0234205 32559214 PMC7304563

[47] Aasgaard HS, Fagerstrom L, Landmark B. Nurses’ experiences of providing care to dementia patients through home health care: after further training and a reorganization of nursing resources. Home Health Care Management & Practice. 2014;26(4):230–8.

[48] Larsen LS, Normann HK, Hamran T. Continuity of home-based care for persons with dementia from formal and family caregivers’ perspective. Dementia. 2019;18(3):846–63. doi: 10.1177/1471301216682626 27927945

[49] Sutcliffe C, Davies K, Ahmed S, Hughes J, Challis D. Delivering Personalised Home Care for People with Dementia: An Investigation of Care Providers’ Roles and Responsibilities. Journal of Long-Term Care. 2021.

[50] St-Amant O, Ward-Griffin C, DeForge RT, Oudshoorn A, McWilliam C, Forbes D, et al. Making care decisions in home-based dementia care: why context matters. Canadian Journal on Aging/La revue canadienne du vieillissement. 2012;31(4):423–34. doi: 10.1017/S0714980812000396 23217659

[51] Peckham A, Williams AP, Neysmith S. Balancing formal and informal care for older persons: how case managers respond. Canadian Journal on Aging/La Revue canadienne du vieillissement. 2014;33(2):123–36. doi: 10.1017/S0714980814000105 24780306

[52] Brodkin EZ. Reflections on street‐level bureaucracy: past, present, and future. Wiley Online Library; 2012.

[53] Schaller S, Mauskopf J, Kriza C, Wahlster P, Kolominsky‐Rabas PL. The main cost drivers in dementia: a systematic review. International journal of geriatric psychiatry. 2015;30(2):111–29. doi: 10.1002/gps.4198 25320002

[54] Reckrey JM, Watman D, Tsui EK, Franzosa E, Perez S, Fabius CD, et al. “I Am the Home Care Agency”: The Dementia Family Caregiver Experience Managing Paid Care in the Home. International Journal of Environmental Research and Public Health. 2022;19(3):1311. doi: 10.3390/ijerph19031311 35162335 PMC8834786

[55] Harris DP, Chodosh J, Vassar SD, Vickrey BG, Shapiro MF. Primary care providers’ views of challenges and rewards of dementia care relative to other conditions. Journal of the American Geriatrics Society. 2009;57(12):2209–16. doi: 10.1111/j.1532-5415.2009.02572.x 19943831 PMC3832192

[56] Sutherland N, Wiersma E. Three Values That Should Underlie Community-Based Dementia Care Strategies. Healthcarepapers. 2016;16(2):52–6. doi: 10.12927/hcpap.2017.25002 28332966

