# Supplementary material for: Towards defining quality in home care for persons living with dementia
Source: PLoS One. 2022 Sep 13;17(9):e0274269. doi: 10.1371/journal.pone.0274269 (PMC9469964; doi:10.1371/journal.pone.0274269)
Supplement: S1 File — (DOCX) [file pone.0274269.s001.docx]

***Exploring the* *Transition from Hospital to Home***

1. Tell me about a hospital admission and transition back home that you experienced…
   1. *Probe*: As you look back on your stay in the hospital, how were you prepared for the transition home?
   2. *Probe*: How were you involved in the discharge planning?
2. Tell me about your most recent transition home from [hospital name]?
   1. What was it like for you [caregiver]
   2. What role [caregiver] did you play?
   3. Who, if anyone, influenced your experience? [probe: ask them to elaborate, how was their experience influenced, etc.]
3. What needs did you have during the transition process?

*Probe: Educational, informational, relational, management*

- 1. How were these needs met or not met?

***Exploring the Adjustment to Being at Home***

1. Can you tell me more about your role as [NAME OF PATIENT]’s caregiver?
2. What was life like once you returned home from the hospital?
3. Did your role as caregiver change for [NAME OF PATIENT] once he/she returned home?

*Probe: How were you prepared? How did you feel?*

1. Tell me about coordinating care since being discharged?

*Probe: How has it been arranging appointments, setting up and accessing services?*

***Discussing Supports and Recommendations***

1. What were some resources or supports that you were able to access?
2. What, if anything, would have made things easier (e.g. family/friends, community, medical)?

**Ending Questions**

Is there anything else that you would like to comment on or share about your transition home?

*Thank you very much for your time and the information you shared today.*
